# Mammalian Frataxin: An Essential Function for Cellular Viability through an Interaction with a Preformed ISCU/NFS1/ISD11 Iron-Sulfur Assembly Complex

**DOI:** 10.1371/journal.pone.0016199

**Published:** 2011-01-26

**Authors:** Stéphane Schmucker, Alain Martelli, Florent Colin, Adeline Page, Marie Wattenhofer-Donzé, Laurence Reutenauer, Hélène Puccio

**Affiliations:** 1 Department of Translational Medicine and Neurogenetics, Institut de Génétique et de Biologie Moléculaire et Cellulaire (IGBMC), Illkirch, France; 2 Inserm U596, Illkirch, France; 3 CNRS UMR7104, Illkirch, France; 4 Université de Strasbourg, Strasbourg, France; 5 Chaire de Génétique Humaine, Collège de France, Illkirch, France; Instituto de Ciencia de Materiales de Madrid - Instituto de Biomedicina de Valencia, Spain

## Abstract

**Background:**

Frataxin, the mitochondrial protein deficient in Friedreich ataxia, a rare autosomal recessive neurodegenerative disorder, is thought to be involved in multiple iron-dependent mitochondrial pathways. In particular, frataxin plays an important role in the formation of iron-sulfur (Fe-S) clusters biogenesis.

**Methodology/Principal Findings:**

We present data providing new insights into the interactions of mammalian frataxin with the Fe-S assembly complex by combining *in vitro* and *in vivo* approaches. Through immunoprecipitation experiments, we show that the main endogenous interactors of a recombinant mature human frataxin are ISCU, NFS1 and ISD11, the components of the core Fe-S assembly complex. Furthemore, using a heterologous expression system, we demonstrate that mammalian frataxin interacts with the preformed core complex, rather than with the individual components. The quaternary complex can be isolated in a stable form and has a molecular mass of ≈190 kDa. Finally, we demonstrate that the mature human FXN_81–210_ form of frataxin is the essential functional form *in vivo*.

**Conclusions/Significance:**

Our results suggest that the interaction of frataxin with the core ISCU/NFS1/ISD11 complex most likely defines the essential function of frataxin. Our results provide new elements important for further understanding the early steps of *de novo* Fe-S cluster biosynthesis.

## Introduction

Human frataxin is the protein deficient in Friedreich ataxia (FRDA), a devastating autosomal recessive neurodegenerative disease associated with hypertophic cardiomyopthy, affecting 1/40,000 in the caucasian population [1], [2]. Frataxin is a highly conserved mitochondrial protein from bacteria to humans [2], [3]. Genetic and biochemical studies support a role of frataxin as a multifunctional protein in different iron-dependent mitochondrial pathways [2], [3], through its ability to bind iron *in vitro* [4], [5] and to deliver iron to different acceptors [6], [7]. Moreover, binding of iron can trigger frataxin oligomerization *in vitro*, a process that was proposed to scavenge toxic iron in a bioavailable form and to be essential for frataxin function [8]. Both monomeric and oligomeric forms of frataxin were shown to interact with various potential iron acceptors. Frataxin was shown to interact *in vitro* with ferrochelatase and to provide the iron that is needed in the last step of heme biosynthesis [7], [9]. Frataxin was also proposed to interact with mitochondrial aconitase, a Fe-S-containing protein, providing protection against the disassembly of the Fe-S cluster by facilitating iron transfer to aconitase [10]. Similarly, and more extensively, both monomeric and oligomeric forms of frataxin were proposed to be the iron donor protein for *de novo* Fe-S cluster biosynthesis [6], [11]–[16].

Fe-S clusters are critical prosthetic groups present in proteins involved in essential cellular processes ranging from nuclear genome stability, protein translation to mitochondrial metabolism [17]. Within the past decade, the biogenesis of Fe-S proteins has been extensively studied in bacteria and yeast demonstrating that it is a complex process involving multiple highly conserved components [17], [18]. *De novo* Fe-S cluster assembly, a mitochondrial process in eukaryotes, relies on the assembly of a Fe-S cluster on a scaffold protein (IscU (bacteria), Isu1 (yeast), ISCU (mammalian)) from inorganic iron and sulfur, followed by the transfer of the scaffold-bound Fe-S cluster to the target apoproteins. Both the synthesis and the final transfer to apoproteins require the help of additional proteins. The sulfur is provided through a persulfide intermediate by a pyridoxal phosphate-dependent cysteine desulfurase, IscS in bacteria and the Nfs1/Isd11 complex in eukaryotes, that interacts with the scaffold protein to form a complex in which Fe-S cluster biosynthesis was proposed to occur *in vivo* [19]. The exact function of the eukaryotic protein Isd11 is unknown, but it has been proposed to stabilize Nfs1 through a direct interaction [20], [21]. The bacterial, yeast and human frataxins (CyaY, Yfh1 and FXN, respectively) were shown to interact with multiple core components of the biosynthesis machinery [11]–[16]. Furthermore, *in vitro*, iron loaded human frataxin was shown to deliver iron to ISCU [6]. However, how frataxin interacts with the Fe-S cluster biosynthesis components remains unclear as direct one-to-one interactions with each component were reported (IscS [12], [22], IscU/Isu1 [6], [11], [16] or ISD11/Isd11 [14], [15]). Finally, the iron-donor function of frataxin has recently been challenged as *in vitro* kinetic studies of Fe-S cluster biosynthesis using the bacterial components revealed that CyaY behaves as an iron-dependent inhibitor of Fe-S cluster assembly through a specific interaction with IscS [22]. Additional experiments are thus required to clarify both the function(s) and the molecular network(s) of the frataxin protein.

To obtain a comprehensive insight into the multiple functions of frataxin, we decided to identify and characterize the interactions of human frataxin. In the present work, we show that endogenous mitochondrial ISCU, NFS1, and ISD11 are the main interactors of frataxin. Using complementary *in vitro* and *in vivo* experiments, we infer that the essential function of mammalian frataxin consists in interacting with the preformed ISCU/NFS1/ISD11 complex, forming a ≈190 kDa quaternary complex that can be isolated in a stable form. Finally, we demonstrate that the function of frataxin is sustained *in vivo* by the mature and monomeric form of frataxin.

## Results

### The Fe-S cluster biosynthesis machinery components NFS1, ISCU and ISD11 are the main interactors of mature frataxin

**Figure 1:**
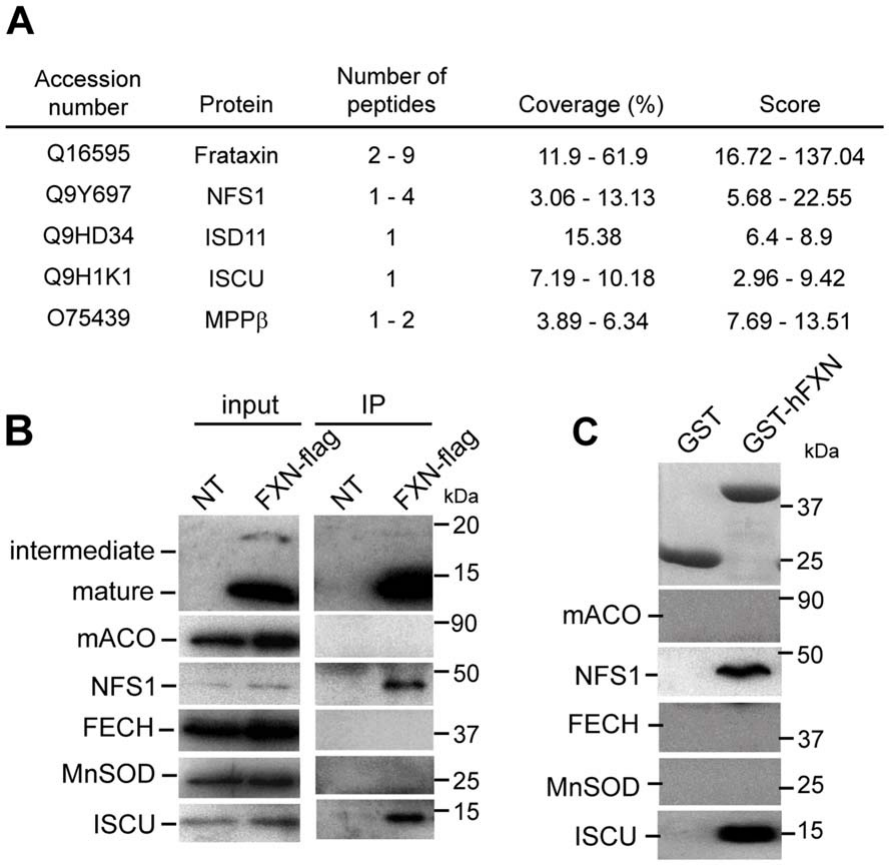
Human frataxin interacts specifically with ISCU, NFS1 and ISD11. (A) Mass spectrometry analysis of proteins identified by co-IP with FLAG-tagged frataxin from HeLa mitochondrial enriched fractions. Results represent the proteins specifically identified common to two independent experiments with hFXN-FLAG expressing cells compared to non-transfected cells. Peptides were selected with a stringent filter to avoid a maximum of false positives. Coverage represents the percentage of sequence matching with peptides found in the analysis. An example of complete results for one experiment is given in Table S1. (B) IP obtained in (A) were analyzed by Western blot using specific antibodies against frataxin (intermediate and mature), mitochondrial aconitase (mACO), NFS1, ferrochelatase (FECH), MnSOD and ISCU. MnSOD was used as a control to evaluate non-specific binding on the beads. Inputs correspond to 5µg of mitochondrial HeLa extracts. (C) GST pull-down using GST-hFXN (aa 81–210) and HeLa mitochondrial extracts. Eluted fractions were analyzed by Western blot as in (B) or by coomassie blue staining to detect GST and GST-hFXN.

We searched for the interacting partners of human frataxin by coupling immunoprecipitation (IP) with mass spectrometry analysis. Due to competition between the immunoprecipitating 1G2 antibody and interactors, IP of endogenous frataxin failed to identify protein partners (data not shown), with the exception of the mitochondrial processing peptidase (MPPβ) involved in the maturation of frataxin [23]. Therefore, IP was performed from HeLa mitochondrial extracts expressing a recombinant human frataxin with a C-terminal flag epitope (hFXN-Flag). As reported, three forms of frataxin, the precursor (25 kDa), the intermediate (19kDa) and the mature (14 kDa) forms [24] were specifically immunoprecipitated from the corresponding HeLa mitochondrial fractions using an anti-Flag antibody (data not shown). Mass spectrometry analysis identified only four common proteins (ISCU, NFS1, ISD11 and MPPβ) from two independent experiments that specifically co-immunoprecipitated with hFXN-Flag, with an average coverage ranging from 3–61% (Fig 1A and Table S1). The four proteins were not detected in the negative control in contrast to the mitochondrial HSPA9 and HSPD1 chaperones (Table S1). The specific interaction with endogenous ISCU and NFS1 was confirmed by western blot analysis (Fig 1B). Unfortunately, the available ISD11 antibody did not detect the endogenous ISD11 from mitochondrial HeLa cell extract.

To further confirm these interactions, we performed GST pull-down experiments from HeLa mitochondrial extracts using the mature form of human frataxin (aa 81–210) fused to an N-terminal GST (GST-hFXN_81–210_). A specific interaction between frataxin and endogenous ISCU and NFS1 was detected by western blot (Fig 1C), in agreement with the IP experiments. Interestingly, the addition of the 1G2 monoclonal antibody, which recognizes an epitope located in the sequence encoded by exon 4 of frataxin, completely abolished the interaction with ISCU and NFS1 (Fig S1A), suggesting a role of this conserved region in the interaction. No difference in interaction with ISCU and NFS1 was detected with the intermediate (GST-hFXN_42–210_) and mature (GST-hFXN_81–210_) forms of frataxin in GST-pull down experiments (Fig S1B), indicating that the site of interaction with the complex is fully comprised within the sequence of the mature form. Therefore, all further experiments were performed with the recombinant mature form of frataxin.

Interestingly, mitochondrial aconitase or ferrochelatase were not detected in the IP and the GST pull-down eluates, despite de presence of both proteins in the soluble input fraction (Fig 1B and 1C). As the interaction between frataxin and mitochondrial aconitase was shown to be promoted by hydrogen peroxide/citrate treatments [10], IP experiments were carried out under these prooxidant conditions. Whether hFXN-Flag or endogenous mitochondrial aconitase were used as bait for IP, no interaction between frataxin and aconitase was observed (Fig S2).

Our results clearly show that the main interactors from mitochondrial HeLa cell extract of a C-terminal or N-terminal tagged human mature frataxin (hFXN_81–210_) are the core components of the *de novo* Fe-S cluster biosynthesis, ISCU, NFS1 and ISD11.

### Frataxin interacts with a preformed ISCU/NFS1/ISD11 complex

**Figure 2:**
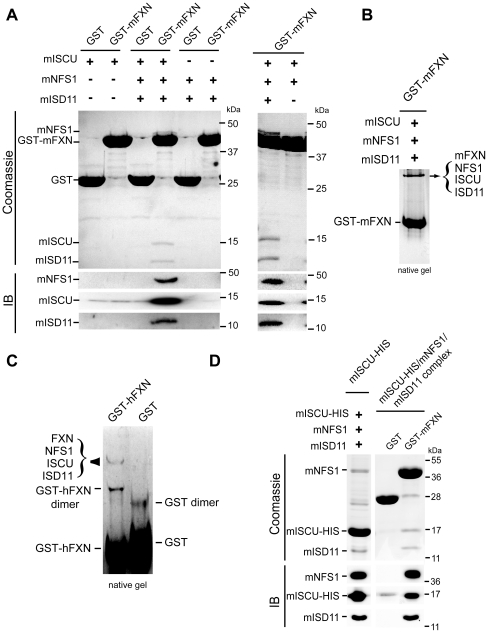
Frataxin binds a pre-formed ISCU/NFS1/ISD11 complex. (A) Co-purification of ISCU/NFS1/ISD11 with GST-mFXN. GST-mFXN or GST were co-expressed with mISCU, mNFS1/mISD11 or mISCU/mNFS1/mISD11 in bacteria (+ and − indicates the presence of each expressing vector). Fractions were analysed by SDS-PAGE and coomassie blue staining (upper panel) or by Western blot (IB). (B) GST-mFXN was co-expressed with mISCU, mNFS1 and mISD11 and purified as in (A). Samples were loaded on a 7.5% non-denaturing gel and stained with coomassie blue. Western blot analysis and mass spectrometry analysis confirmed that the upper band corresponds to a complex containing mFXN, mISCU, mNFS1 and mISD11. (C) Co-purification of NFS1/ISD11 with mISCU-HIS (left). mISCU-HIS was co-expressed with mNFS1 and mISD11 and purified by cobalt column. Co-purification of mISCU-HIS/mNFS1/mISD11 with GST-mFXN (right). GST pull down using GST or GST-mFXN with purified mISCU-HIS/mNFS1/mISD11 complex. The samples were loaded on a SDS-gel and analysed by Western blot using NFS1, ISD11 and ISCU specific antibodies. + and − indicate the presence and the absence of the corresponding vectors, respectively. (D) Native complex from HeLa cells. GST and GST-hFXN were incubated with mitochondrial HeLa extract, pull-down and loaded on non-denaturing gel. Only two protein complexes were detected by coomassie staining. Mass spectrometry analysis and western blot confirmed that one corresponds to GST-hFXN dimer and the second to the GST-hFXN/ISCU/NFS1/ISD11 complex. With the exception of the common contaminating proteins (keratins and elongation factor 1) found by mass spectrometry analysis, the only proteins present in the band corresponding to the quaternary complex were NFS1 (10 peptides, 28.2% coverage), hFXN (5 peptides, 18.6% coverage), ISCU (4 peptides, 24% coverage) and ISD11 (4 peptides, 29.7% coverage).

To determine which component of the ISCU/NFS1/ISD11 complex is the direct partner of frataxin, we co-expressed the mature form of the murine homologues in multiple combinations in the bacteria, followed by GST purification. For all bacterial expressed recombinant proteins (GST or His-tagged), the mitochondrial targeting sequence was removed from FXN, NFS1 and ISCU. Each construct expressed soluble protein in bacteria when expressed alone or in combination (Fig S3A). When GST-mFXN was co-expressed either with mISCU alone or with mNFS1/mISD11 or mNFS1/mISCU, no co-purification was detected (Fig 2A). It is only when GST-mFXN, mISCU, mNFS1 and mISD11 were co-expressed that a co-purification of all four proteins was observed (Fig 2A). These results demonstrate that frataxin is able to interact with co-expressed ISCU, NFS, ISD11 proteins and suggest that frataxin interacts with a ISCU/NFS1/ISD11 complex rather than with individual components. As mNFS1/mISD11 are the limiting factors due to lower expression and insolubility in bacteria compared to GST-mFXN and mISCU (data not shown), little complex was purified compared to the large excess of GST-mFXN. However, when a limiting amount of purified GST-mFXN was used to pull-down the complex from a bacterial extract expressing mISCU/mNFS1/mISD11, the quaternary complex could be isolated at nearly stoichiometric levels (Fig S3B). In the absence of mISD11, neither the quaternary nor the ternary complex formed (Fig. 2A), further suggesting that ISD11 is important for NFS1 stability.

Native PAGE analysis of both bacterial co-purification (Fig 2B) and GST-hFXN pull-down eluates obtained with endogenous ISCU, NFS1 and ISD11 from HeLa mitochondrial extracts (Fig 2C) revealed the presence of one major complex containing only the four proteins as verified by mass spectrometry (Fig. 2C) and western blot (data not shown), further demonstrating the presence of a quaternary complex. The identification of endogenously expressed ISD11 in the native complex further confirmed the original co-IP experiments. When isolated from bacterial co-expression, the quaternary complex could be easily concentrated to 5µg/µl, without precipitation.

While frataxin only interacts in a quaternary complex, the ternary complex mISCU/mNFS1/mISD11 could be isolated in the absence of frataxin. Indeed, when mISCU-HIS, mNFS1 and mISD11 were co-expressed, mNFS1 and mISD11 co-purified with mISCU-HIS (Fig 2D). Furthermore, pull-down experiments using GST-mFXN as a bait demonstrated an interaction between GST-mFXN and the pre-purified mISCU-HIS/mNFS1/mISD11 complex (Fig 2D). In contrast to the quaternary complex, the ternary mISCU/mNFS1/mISD11 complex was susceptible to aggregation upon concentration.

Contrary to the reported data in yeast demonstrating that iron reversibly increase the binding of Yfh1 to Isu1/Nfs1 [11], under similar experimental conditions, we did not observe any increase nor decrease of FXN binding to the core complex (Fig S4A–B). Furthermore, high (100µM) or low (using bathophenanthroline Fe^2+^ chelation) iron concentrations in reducing condition, or the presence of different metals (50 µM Ca^2+^, Ni^2+^, Zn^2+^, Mg^2+^) had no effect on the formation of the complex (Fig S4C–D).

Together, our results demonstrate that frataxin interacts with a preformed ISCU/NFS1/ISD11 complex rather than with the individual components to form a stable quaternary complex.

### A surface encompassing the acidic ridge and the b-sheets is involved in the interaction between frataxin and the core complex

**Figure 3:**
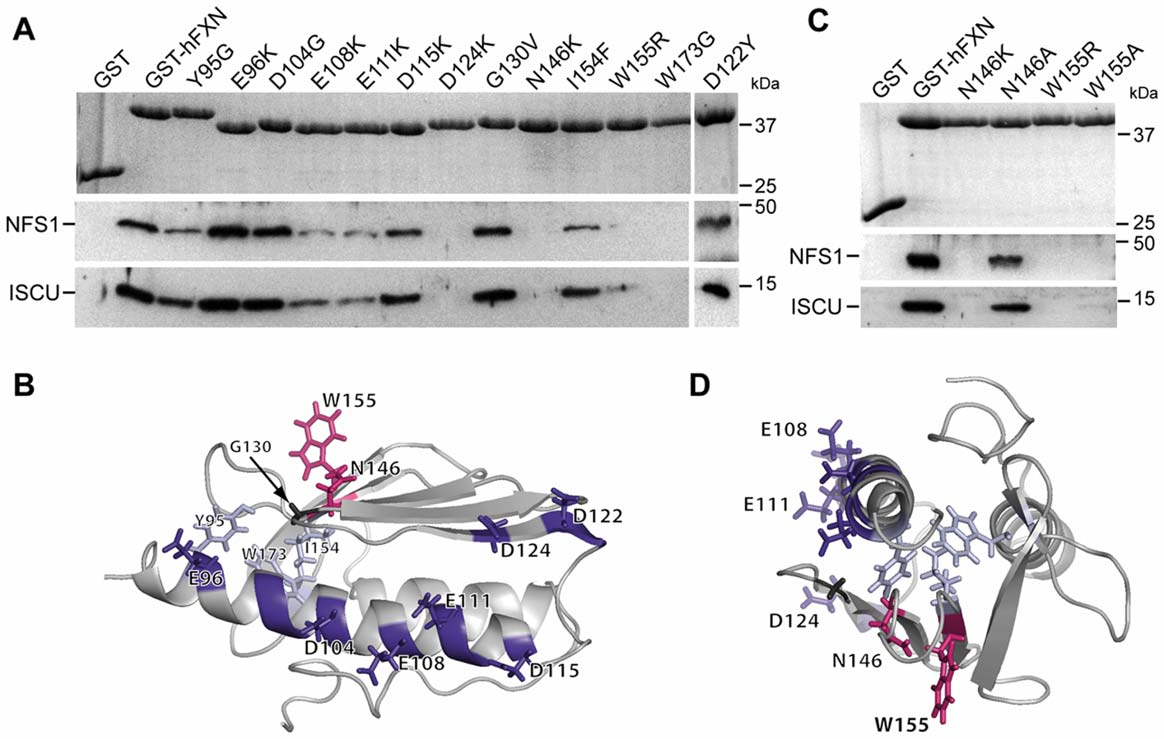
Residues in the αa1-helix and the β-sheets of frataxin are crucial for the interaction with ISCU and NFS1. (A) GST pull-down using different GST-hFXN mutants. GST-hFXN mutants were obtained by directed mutagenesis (only amino acid changes are indicated). GST pull-downs were carried out as in Fig 1C and analyzed by SDS-PAGE and coomassie blue staining to visualize GST-hFXN (upper panel), and by Western blot using antidodies against NFS1 and ISCU. (B) Solution structure of human frataxin (PDB ID 1LY7) showing the localization of the residues mutated. (C) GST pull-down using GST-hFXN mutants N146K, N146A, W155R and W155A. Experiments were carried out as in Fig 1C. (D) Top view of the solution structure shown in (B). The five residues affecting the interaction with the ISCU/NFS1/ISD11 complex define a potential interaction surface on frataxin.

To identify residues on frataxin that are essential for the interaction with the ISCU/NFS1/ISD11 complex, 13 recombinant GST-tagged human mature FXN_81–210_ carrying specific point mutations were tested by GST pull-down using mitochondrial HeLa cell extracts (Fig 3A–B). Again, only endogenous ISCU and NFS1 could be detected by western blot due to the inability of the ISD11 antibody to detect endogenous ISD11. Six of the mutational substitutions constructed are pathological mutations found in compound heterozygous FRDA patients (D122Y, G130V, N146K, I154F, W155R and W173G) [25]. Seven mutations were directed against acidic residues from the acidic ridge of frataxin (E96K, D104G, E108K, E111K, D115K, D122Y and D124K). One mutation, Y95G, was proposed to stabilize the trimeric structure in yeast frataxin [26]. All mutations involved residues exposed on the protein surface except Y95, I154 and W173, for which side chains are mostly buried in the protein core (Fig 3B). Therefore, the absence of interaction of the W173G recombinant protein and the reduced interactions of the Y95G and I154F recombinant proteins most likely reflect a structural role of these residues (Fig 3A). These results are consistent with several studies that have reported the effect of the W173G and I154F mutations on the stability of the human frataxin protein, both *in vitro* and *in vivo* [15], [27]–[29]. While the E108K and E111K mutants enabled the interaction with the complex, although diminished, the D124K mutation caused a drastic decrease in the interaction with ISCU and NFS1 (Fig 3A). Mutations at the acidic residues E96, D104, D115 and D122, as well as the pathological G130V mutation, associated with an atypical milder phenotype in FRDA, had no impact on the interaction (Fig 3A). In contrast, the W155R and the N146K mutations strongly affected the interaction with NFS1 and ISCU (Fig 3A). Similarly, ISCU, NFS1 and ISD11 were not detected by mass spectrometry analysis (Table S1) or western blot in a FXN^N146K^-flag IP from HeLa mitochondrial extract, although MPPβ was detected (data not shown). Furthermore, no co-purification of mNFS1, mISCU and mISD11 could be observed using GST-hFXN^N146K^ in a bacterial co-expression experiment (Fig S3C).

As the absence of interaction with the N146K and W155R mutants could be due to steric or electrostatic hindrance, two milder mutations were constructed, replacing N146 and W155 by alanines. The N146A mutation restored the interaction with the complex, while the W155A mutation did not (Fig 3C), indicating that the protruding tryptophan is an essential residue for interaction with the complex.

We have therefore identified three mutations directed against the residues from the acidic ridge of frataxin (E108K, E111K and D124K) and two surface-exposed mutations (W155R and N146K) that specifically disrupted the interaction of frataxin with the core complex, defining a surface of interaction on one side of frataxin, spreading from the acidic ridge to the β4-sheet (Fig 3D).

### The association of frataxin with the *de novo* Fe-S cluster biosynthesis complex is crucial for cellular function

**Figure 4:**
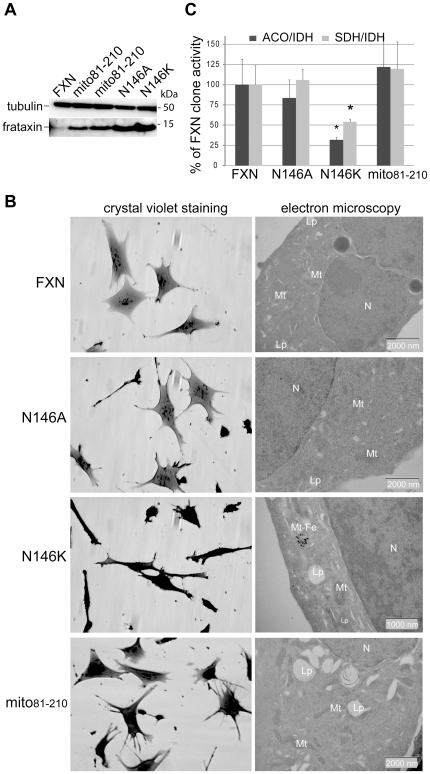
The interaction between frataxin and the Iscu/Nfs1/Isd11 complex is essential for cellular function. (A) Western blot analysis of mitochondria-enriched fractions from clones expressing wild type frataxin, mito81–210, FXN^N146A^ and FXN^N146K^ using anti-frataxin and anti-tubulin antibodies. (B) Morphological and ultrastructural alterations in FXN, N146A, N146K and mito81–210 clones. Each clone was studied by phase contrast microscopy after crystal violet staining (left panels) and electron microscopy analysis (right panels). mt, mitochondria; Lp, lipid droplet; mt-Fe, intramitochondrial iron deposits; N, nucleus. (C) Biochemical measurements of Fe-S enzyme activities in FXN, N146A, N146K and mito81–210 clones. Succinate dehydrogenase (grey bars) and aconitases (dark grey bars) specific activities were standardized to isocitrate dehydrogenase (IDH) specific activity and expressed as percentage of control activity. Results were obtained from two independent experiments using 4 FXN, 3 N146A, 1 N146K and 4 mito81–210 clones. Data are represented as mean + SD. * p<0.05.

To determine whether the interaction between frataxin and the core complex is an essential function of frataxin *in vivo*, we generated four murine fibroblast cell lines stably expressing full length hFXN^N146K^, hFXN^N146A^, hFXN^W155R^ or hFXN^W155A^ in the conditional allele background enabling the deletion of the endogenous gene [30]. Complete frataxin deficiency in this fibroblast cell line does not sustain cell division and survival [30]. All mutant proteins were properly targeted to mitochondria and matured (Fig S5A). After endogenous frataxin deletion, while we isolated 12 clones carrying wild-type frataxin, 5 clones expressing the hFXN^N146A^, and a single clone expressing the hFXN^N146K^ (Fig 4A), no clone expressing either hFXN^W155R^ or hFXN^W155A^ was isolated (Fig S5B–C), further demonstrating that W155 is an essential residue for frataxin function. Wild-type and N146A clones did not present any gross phenotype (Fig 4B), nor sensitivity to oxidative stress (Fig S5C–D), a feature of frataxin haploinsufficiency [30]. In contrast, the N146K clone showed spindle-shaped and retracted cells with a strong growth defect, and displayed the classical features of FRDA (degenerating mitochondria, electron dense deposits, deficit in Fe-S enzyme activities) (Fig 4B–C and Fig S5E). Together, these results strongly suggest that interfering with the quaternary complex formation in the cell leads to a typical FRDA phenotype and that the interaction of frataxin with the core complex is the essential function of mammalian frataxin *in vivo*.

### The quaternary complex has a molecular mass of ≈190 kDa

**Figure 5:**
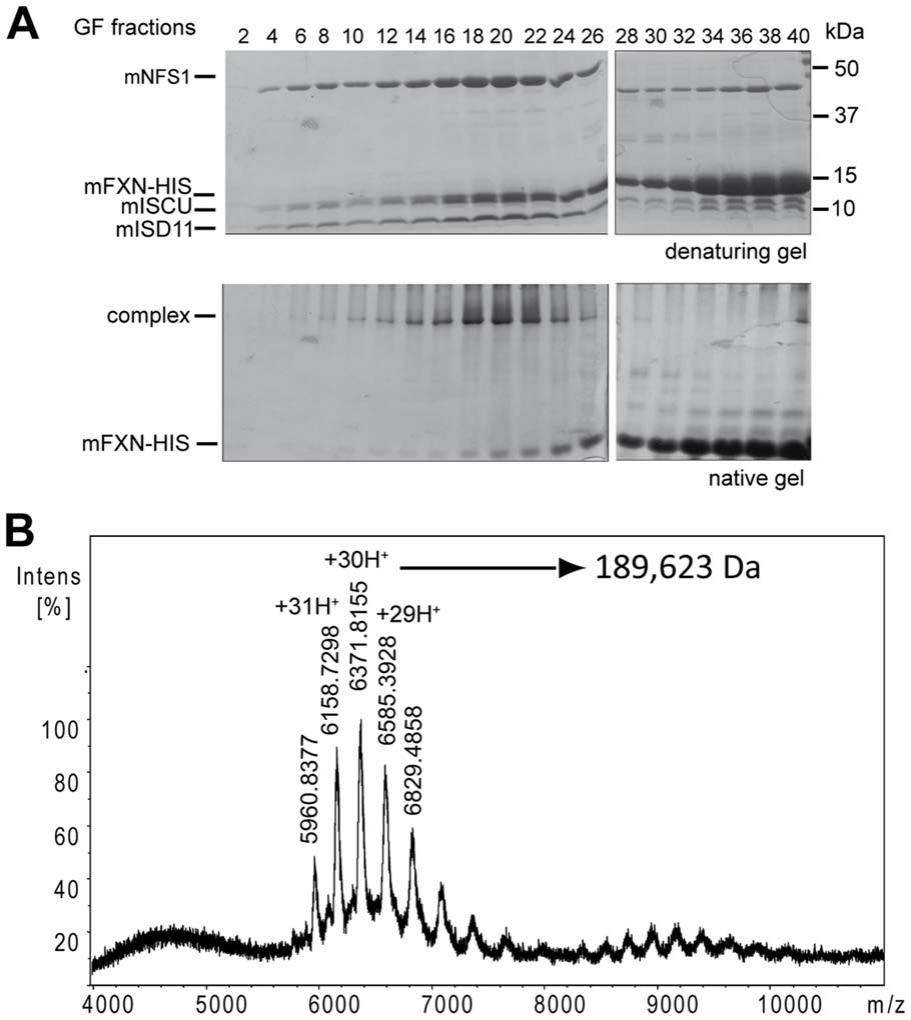
Determination of the molecular weight of the FXN/ISCU/NFS1/ISD11 complex. (A) Gel filtration for the mFXN-HIS/mISCU/mNFS1/mISD11 complex. After a single nickel column purification, the sample was loaded and separated by gel filtration. Coomassie staining of a denaturing and a non-denaturing gel corresponding to fractions 2 to 40 are shown. Fractions 19 and 20 that contained the complex were concentrated for the native mass spectrometry analysis. (B) ESI native mass spectra of the mFXN-HIS/mISCU/mNFS1/mISD11 complex after gel filtration purification. The experimental molecular weight for each component was 10,722 Da, 14,543 Da, 15,333 Da and 44,669 Da for mISD11, mISCU, mFXN-HIS and mNFS1, respectively. The mass was estimated to the molecular weights of 189,623 Da.

Two different strategies were followed to purify the quaternary complex. Either a double GSH and nickel column purification or a single nickel column purification was carried out from GST-mFXN/mISCU/HIS-mNFS1/mISD11 or mFXN-HIS/mISCU/mNFS1/mISD11 expressing bacteria, respectively (Fig S6A). The complexes were further purified by gel filtration. Fractions containing the GST-mFXN/mISCU/HIS-mNFS1/mISD11 complex (fractions 14 to 18 corresponding to ≈4 mg of complex, Fig. S6B) or the mFXN-HIS/mISCU/mNFS1/mISD11 (fractions 19 and 20 corresponding to ≈3 mg of complex, Fig. 5A) were concentrated for native ESI mass spectrometry analysis. Multicharged ions were detected for both complexes, even at high potential (250 kV) (Fig 5B and Fig S6C). Molecular weights of 237,130 Da and 189,623 Da were deduced for the GST- mFXN/mISCU/HIS-mNFS1/mISD11 and mFXN-HIS/mISCU/mNFS1/mISD11 complexes, respectively. The difference between the masses of the two complexes is suggestive of two frataxins per complex, considering the difference in mass between GST-mFXN and mFXN-HIS (Fig S6D). Furthermore, using a concentration curve of purified proteins, the mISCU∶mFXN ratio per complex was estimated to be one (Fig S6E). Such calculation could however not be achieved for mNFS1 or mISD11, as neither protein is stable when expressed alone. Although the ternary complex can be isolated by gel filtration (Fig S7A), the yield is very low due to instability of the complex upon concentration. As for the bacterial IscU/IscS complex [19], the ternary complex can be stabilized by the insertion of the D72A mutation on mISCU (Fig S7B). However, this stabilization is not sufficient to allow concentration for ESI mass spectrometry analysis. Therefore, assuming mNFS1 as a dimer, the observed mass is suggestive of stoichiometric amount of mNFS1, mFXN and mISCU. Interestingly, the mass is compatible with the presence of 3 or 4 mISD11 per complex (Fig. S6D).

### The mitochondrial hFXN_81–210_ form of frataxin is the essential functional form *in vivo*

Mature hFXN_81–210_ is the main endogenous form of frataxin in human tissues and cell lines [24]. However, during the maturation process, a longer intermediate form (hFXN_42–210_) is expressed. This intermediate form, as well as a longer hFXN_56–210_ form can interact with the core complex (Fig S1B). To determine whether hFXN_81–210_ is the essential functional form of frataxin *in vivo*, we expressed a recombinant protein (mito_81–210_) with its own mitochondrial targeting sequence (aa 1–41) fused directly to the sequence of mature frataxin (aa 81–210), bypassing the intermediate hFXN_42–210_ form. Interestingly, when expressed in Cos-1 cells, although mito_81–210_ was correctly processed, the level of mature frataxin was decreased, and the precursor form accumulated within the cytosol (Fig S5F), suggesting that amino acids 42–80 of human frataxin are partially involved in the mitochondrial targeting. In murine fibroblasts, mito_81–210_ was able to completely rescue the cell lethality induced by endogenous frataxin deletion, whereas a cytosolic version of hFXN_81–210_ was unable (Fig 4 and Fig S5B–C). The clones expressing mito_81–210_ showed no morphological or structural abnormalities (Fig 4B), normal Fe-S enzyme activities (Fig 4C), and no susceptibility to exogenous oxidative stress (Fig S5D). These results demonstrate that mito_81–210_ is sufficient to complement endogenous frataxin functions and promote survival, providing the evidence that mature hFXN_81–210_ is the functional form for *in vivo* Fe-S cluster biosynthesis.

## Discussion

Frataxin has been proposed to be a multifunctional protein based on its numerous interactions, including components of the Fe-S biosynthesis machinery, ferrochelatase, mitochondrial aconitase, succinate dehydrogenase, and several chaperones [2], [9]–[12], [15]. In the present study, the only endogenous proteins that co-purified with a recombinantly expressed frataxin are ISCU, NFS1, ISD11 and MPPβ, suggesting that these proteins stably associate with frataxin, and belong thus to the core network of frataxin partners *in vivo*. Furthermore, we showed that *in vivo* expression of frataxin mutants that affect the interaction with the core components of *de novo* Fe-S cluster biosynthesis triggers cell death (W155R and W155A) or a classical FRDA phenotype (N146K), while frataxin mutants that keep the ability to interact lead to normal cellular phenotype (N146A). Altogether, our results strongly suggest that the interaction of frataxin with the Fe-S cluster assembly complex defines the essential function of frataxin *in vivo*. Although the relevance of the other reported interactions needs to be further investigated *in vivo*, we cannot exclude that less stable or transient interactions with frataxin could not be detected in our experimental conditions. Furthermore, we show that mitochondrial mature frataxin (hFXN_81–210_) was sufficient to support cell survival with a normal phenotype (Fig 4).

Each of the individual components of the *de novo* Fe-S biosynthesis complex (Isu/Iscu, IscS and Isd11) has been proposed to be the molecular adaptor for frataxin interaction [6], [11], [12], [14], [15], [22]. However, in the heterologous expression system, our results demonstrate that mammalian frataxin interacts with the preformed ISCU/NFS1/ISD11 complex rather than with the individual proteins. The interaction of frataxin in complex with both the Fe-S scaffold and the cysteine desulfurase is most likely conserved throughout evolution, as demonstrated by the co-purification of Isu/Nfs1 with Yfh1 in yeast [11] and IscU/IscS with CyaY in bacteria [12]. However, in contrast to our results with the mammalian protein, in yeast, a N122K mutation on Yfh1 (equivalent to the human N146K) had more effect on the interaction with Isu1 than with Nfs1/Ids11 [16], indicating that this residue is most likely involved in the interaction with the scaffold protein. In addition, mutations on positively charged residues of IscS were recently reported to affect specifically the interaction of IscS with CyaY [31]. Similarly, mutations on positively charged residues of Nfs1 perturbed the complex formation in the mammalian system (Fig S3D). Together with our results, these data strongly suggest that NFS1 and ISCU define the interaction surface that is targeted by frataxin. Based on the mutational screen results and the published literature [13], [15], [16], [22], [31], we propose that part of the acidic ridge of frataxin could be involved in the interaction with conserved basic residues of NFS1, while residues on the β-sheets (including W155) would be crucial for the interaction with ISCU. Although π interactions between the W155 and R165 residues contribute to frataxin stability [28], it is unlikely that a fold defect of the W155A mutant underlies the loss of interaction with the complex, as the protein was properly expressed and matured *in vivo*, and was not degraded by the mitochondrial quality control protease machinery. Furthermore, in Yfh1, a R141A mutation which should elicit the loss of the π interactions between W131 and R141, had no effect on the interaction with Isu1 [13]. Finally, the crystal structure of the bacterial IscS/IscU complex demonstrated one IscU monomer per active site of IscS, which are located on opposite sides of the IscS homodimer [31]. Based on the stoichiometry and the mutagenesis experiments, it is reasonable to hypothesize that there is one FXN monomer and one ISCU monomer per active site of NFS1. Furthermore, the mass spectrometry data suggest that there are 3–4 ISD11 on the complex. While the exact role of the eukaryotic ISD11 is not known, it is clearly essential for both the quaternary and ternary complexes formation (Fig 2A). Whether frataxin and ISD11 have direct interacting surfaces still needs to be determined.

It has recently been suggested that iron oxidation-driven oligomerization of yeast frataxin promotes the assembly of a stable core Fe-S complex [14]. Our data clearly demonstrate that the mature hFXN_81–210_ is the essential form of frataxin *in vivo*. While we have not directly tested the capacity of hFXN_81–210_ to oligomerise, it has recently been reported that hFXN_81–210_ is not prone to iron-induced oligomerisation [32], in contrast to yeast or bacterial frataxin or longer forms of human frataxin. Furthermore, our native gel analyses as well as our gel filtration and stoichiometric data are incompatible with an oligomeric form of frataxin as the functional unit in the purified quaternary complex. Finally, it is worth noting that the sites of interaction with the complex that we have identified on frataxin are buried within the oligomeric structure [26]. Together, these results are not supportive of an oligomeric form of frataxin as the essential functional unit *in vivo* for Fe-S cluster biosynthesis, but rather suggest that mature frataxin is monomeric.

Frataxin has been proposed to be either the iron donor for Fe-S cluster biosynthesis or a regulatory protein that inhibits Fe-S cluster formation [11], [22]. Although we cannot conclude on the role of frataxin as the iron donor for Fe-S cluster assembly, our experiments indicate that the iron concentration does not modulate the interaction between mammalian frataxin and the ternary complex. While the reason for this observed difference with the yeast remains to be determined, it might arise from the relatively different iron-binding properties of mammalian frataxin in comparison to yeast frataxin [3]. Furthermore, *in vivo* frataxin deficiency [33], [34], [35] as well as the expression of the N146K mutant (Fig 4C) lead to a strong decrease of Fe-S cluster biosynthesis rather than a complete deficit. These data demonstrate that frataxin is required but not essential for proper Fe-S cluster biosynthesis, and suggest that frataxin might facilitate efficient Fe-S cluster biosynthesis *in vivo*. In bacteria, cluster assembly has been demonstrated to be a dynamic process that involves the dissociation of IscU and IscS for cluster transfer [19]. By interacting with a preformed ISCU/NFS1/ISD11, FXN probably prevents the dissociation of NFS1/ISD11 and ISCU, having the effect of increasing the stability of the ISCU/NFS1/ISD11 interaction. To quantitatively demonstrate the effect of frataxin on the stability of the ternary complex, the affinities of ISCU for NFS1/ISD11 in the absence and the presence of frataxin should be measured. However, the current technical difficulties to purify large quantities of the mammalian NFS1/ISD11 complex prevent such an approach. Assuming that, as mammalian frataxin, CyaY preferentially interacts with the preformed IscU/IscS complex, the addition of frataxin in reconstitution experiments would lead to uncompetitive inhibition, thus explaining the reported inhibitory effect of CyaY on Fe-S cluster formation *in vitro* [22].

In conclusion, the properties of the interaction of frataxin with the core complex in the heterologous system open new perspectives on how frataxin might facilitate Fe-S cluster biogenesis *in vivo*. While in the absence of frataxin, the interaction between ISCU and NFS1/ISD11 is unstable and very dynamic [19], frataxin might function by maintaining the ISCU/NFS1/ISD11 interaction. Interestingly, a recent crystal structure of the bacterial IscS/IscU heterotetramer demonstrated that the catalytic cysteine of IscS and the acceptor cysteines on IscU are too far in distance to allow direct transfer, suggesting that a conformational rearrangement is necessary to bring together the sulfur donor and the acceptor cysteines [31]. It is tempting to speculate that frataxin interaction with the complex triggers this conformational rearrangement to allow efficient sulfur and iron transfer. By analogy to *Thermus Thermophilus* Nqo15, the frataxin-like subunit present in the hydrophilic domain of the respiratory chain complex I [36], one possibility would be that frataxin creates a channel on the interface with the complex allowing a more efficient iron delivery into the site of cluster synthesis. It has recently been proposed, based on biochemical and biophysical data, that the Isu1-interacting surface of yeast frataxin encompasses the iron binding sites, thereby positioning frataxin for iron delivery to Isu1 close to the Fe-S cluster assembly site [37]. The authors suggest a model in which frataxin would dissociate from the complex following iron delivery, prompting Isu1 to complete Fe-S cluster assembly [37]. On the other hand, a stabilised complex might be necessary to provide a safe environment for Fe-S cluster formation or to allow all the consecutive reaction steps to occur. Further challenging biochemical and spectroscopic studies are necessary to characterize the role of frataxin within the quaternary complex, as well as the sequence of events of the early steps of *de novo* Fe-S cluster biogenesis.

While our manuscript was under review, two groups independently reported biochemical results that nicely complement our work [38], [39]. Similarly to our results, Tsai *et al.* provided *in vitro* evidence that human frataxin binds, in an iron independent manner, to a NFS1, ISCU and ISD11 complex to generate the quaternary complex [38]. Very interestingly, they demonstrated that frataxin stimulates the cysteine desulfurase activity. As the cysteine desulfurase activity could be further stimulated by the additon of Fe^2+^, the authors proposed that frataxin functions with Fe^2+^ as an allosteric activator for Fe-S cluster assembly. In the second manuscript, by combining small-angle X-ray scattering (SAXS) and NMR studies with mutagenesis studies on the bacterial homologs, Prischi *et al.* have defined an interaction surface between CyaY, IscS and IscU that is consistent with the interaction surface that we have proposed [39]. Furthermore, through biolayer interferometry, they demonstrated that CyaY enhances the affinity of IscU on IscS, thereby stabilizing the complex, providing the biophysical evidence for the inhibitory effect of CyaY on IscS/IscU enzymatic kinetics. In the future, it will be extremely interesting to further compare the biophysical characteristics of the complexes from different organisms and to determine the effect of the pathological FRDA mutations on the cysteine desulfurase activity and Fe-S cluster assembly.

## Materials and Methods

### Recombinant protein purification

The technical procedures involved in plasmid and strain constructions are described in Material and Methods S1. For low scale GST purifications, the suspension was centrifuged 15,000 g, 4°C for 15–30 min and the supernatant was incubated with glutathione-S-sepharose beads (Pharmacia) for 3 hours at 4°C. The mix was loaded on chromatography column (BIORAD) and washed with PBS. Elution was performed with 35mM glutathione in Tris-HCl 100mM, pH 8, NaCl 150mM for 10 min at 4°C. Elutions were dialysed 2–3 hours in PBS, 5mM EDTA, 5mM DTT at 4°C using dialysis column (ThermoScientific). For large-scale purification, the suspension was centrifuged 40,000 rpm (Ti50.2, Beckman), 4°C, 30 min and the supernatant was further purified by FPLC (AKTA, GE Healthcare). The supernatant was loaded on Cobalt or Nickel affinity column (GE Healthcare) or glutathione sepharose affinity column (GE Healthcare). After wash with PBS, elution was carried out using 200mM imidazole or 35mM glutathione. Gel filtration (GF) was carried out on Superdex S200 (16/90) or (10/30) (Amersham Biosciences) columns. For native mass spectrometry analysis, elution was performed with 50mM ammonium acetate. Samples were concentrated using Vivaspin columns (c.o. 100,000, Sartorius). Protein concentrations were determined by Bradford assay.

### GST pull-down assays

GST pull-downs were performed by incubating 100µg of GST-fused protein with 5–10mg mitochondria enriched extracts and glutathione-S sepharose beads for 3 hours in Tris-HCl 100mM, pH 7.5, 10% glycerol, 100mM KCl and Complete protease inhibitor cocktail (Roche). The mix was loaded on a chromatography column (BIORAD), washed with PBS, 100mM KCl. Elution was performed in loading buffer after boiling for 10 min. To test the effect of metals on the interactions, 50µM or 100µM FeSO_4_ with 500µM-10mM ascorbic acid or 50 µM of either NiSO_4_, MgSO_4_, CaCl_2_ or ZnCl_2_ were added during extraction and wash steps. Iron chelators (EDTA or bathophenanthroline disulfonic acid) 1mM were added in some samples during wash or incubation steps, as indicated in figure legends.

### Cell culture and protein extraction

HeLa cells (ATCC-USA) were grown and transfected as previously described [24]. Cells were harvested 24 hours after transfection. Culture, stable transfection, and clonal selection of immortalized mouse fibroblasts established from *Frda^L3/L−^* mice were carried out as described [30]. Total, mitochondrial-enriched and cytosolic fractions were obtained as described [24].

### Biochemical assays

Enzymatic activities, crystal violet staining, electron microscopy and determination of the sensitivity to stress were performed as previously described [30].

### Immunoprecipitation

FLAG-tagged proteins were immunoprecipitated by incubating overnight 100–200µL FLAG M2 coupled resin (SIGMA) (prepared as recommended by the manufacturer) with 500µg to 1.5mg of mitochondria enriched extract in Tris-HCl 100mM, pH 7.5, 10% glycerol, 100mM KCl and Complete protease inhibitor cocktail (Roche). The mix was loaded on a chromatography column (BIORAD), washed 4 times with 5 mL PBS, 100mM KCl. Elution was performed by resuspending the beads in loading buffer and boiling for 10 min or by incubating beads with glycine 0.1 M pH 3 for 10 min. IP of endogenous mitochondrial aconitase was performed as described [10].

### PAGE and Western blot analysis

14% acrylamide SDS-Glycine-PAGE was used unless otherwise indicated. 7.5% or 10% acrylamide native gels were prepared without reductant. Western blot were carried out as previously described [24]. Antibodies were diluted as indicated in Material and Methods S1. Silver staining was performed as described [24]. Quantification of coomassie blue staining was performed on a Chemigenius^2^ (SYNGENE).

### Mass spectrometry analysis

IP were loaded on 4–12% NuPAGE® gel (INVITROGEN). Ten bands from 5 kDa to 130 kDa were cut and prepared for MS analysis using trypsin for the protein digestion. Samples were analysed by NanoLC-nanoESI/MS^2^. Native mass determination was carried out by ESI-MS analysis. Further details are given in Material and Methods S1.

### Statistics

All data represent mean ± SD. The statistical analysis was performed by standard Student T-test. Statistical significance was considered at p<0.05.

## Supporting Information

Figure S1 **Conserved domain of frataxin is involved in the interaction with ISCU and NFS1.** (A) The monoclonal 1G2 antibody directed against exon 3–4 of frataxin prevented the interaction between frataxin and ISCU and NFS1. GST pull-down was performed as described in Fig. 1C with the addition (+) or not (−) of 1G2 antibody. Note that the 1G2 antibody is retained by GST-hFXN (heavy and light chain) and prevents interaction with ISCU and NFS1. (B) GST-hFXN pull-down was performed on mitochondrial Hela extract as in Fig. 1C using different GST-hFXN constructs. As the capacity of GST-hFXN_42–210_, GST-hFXN_56–210_ and GST- hFXN_81–210_ to interact with NFS1 and ISCU were equivalent, we perform all following GST pull-down with the GST-hFXN_81–210_ expressing construct that corresponds to the size of the endogenous mature human frataxin.
(JPG)

Figure S2 **Absence of interaction between frataxin and mitochondrial aconitase.** (A) Immunoprecipitation of mitochondrial aconitase from HeLa mitochondrial extracts was performed exactly as described (1), with the same anti-aconitase antibody kindly provided by Anne-Laure Bulteau. Mitochondria were treated with 100 µM hydrogen peroxide and 2 mM of citrate (+) prior to the IP. Samples were loaded on SDS-PAGE and analysed by Western blot using specific antibodies against frataxin and mitochondrial aconitase. (B) Immunoprecipitation of hFXN-FLAG was performed as in Fig. 1A. Mitochondria were treated or not with hydrogen peroxide and citrate as in (A). Samples were analysed by Western blot.
(JPG)

Figure S3 **Frataxin interaction with ISCU/NFS1/ISD11 complex and effects of mutations.** (A) Expression of mNFS1, mISCU and mISD11 in bacteria co-transformed with different sets of vectors. Soluble fractions of bacteria expressing GST-FXN, mNFS1, mISD11 and/or mISCU were loaded on a SDS-gel and analysed by Western blot using NFS1, ISD11 and ISCU specific antibodies. + and − indicate the presence and the absence of the corresponding vectors, respectively. (B) GST pull-down using a limiting amount of GST-FXN and a bacterial extract expressing mISCU/mNFS1/mISD11. 25mg of GST or GST-FXN were added to the bacterial lysate before purification on glutathione-sepharose beads. The elutions were analyzed on SDS-PAGE by silver staining. (C) Co-purification of ISCU/NFS1/ISD11 with GST-hFXN^N146K^. GST-hFXN or GST-hFXN^N146K^ (N146K) were co-expressed with mISCU, mNFS1 and mISD11 and purified on glutathione-S-sepharose column as in Fig. 2A. Elutions were analysed by SDS-PAGE and coomassie blue staining (upper panel) or Western blot (IB). (D) Mutations of positively charged residues on NFS1 affect frataxin interaction with the complex. R220D and R225D mutations were introduced by directed mutagenesis on mNFS1 cDNA. Co-purification was carried out as in Fig. 2A and analyzed on native and denaturing gel by coomassie blue staining. Western blot on mNFS1 was performed to verify the correct expression of the two mNFS1 mutants.
(JPG)

Figure S4 **Absence of effect of different metals on complex formation.** (A) Effect of iron on the interaction of frataxin with ISCU and NFS1. GST pull-down was carried out as in Fig. 1C with the exception that the extraction, purification and wash steps were carried out in the absence or the presence of 100 mM FeSO_4_/1 mM ascorbate (Fe^2+^) and 1 mM EDTA as indicated. (B) Effect of iron on native GST-mFXN/ISCU/NFS1/ISD11 complex. GST-mFXN was co-expressed with mISCU, mNFS1 and mISD11 and purified as in Fig. 2*A*. Iron or EDTA was added during extraction and wash steps. Samples were loaded on a 7.5% non-denaturing gel and stained with coomassie blue. Western blot analysis and mass spectrometry analysis confirmed that the upper band corresponds to a complex containing mFXN, mISCU, mNFS1 and mISD11. (C) Effect of iron on the complex formation. A bacterial soluble extract containing mNFS1, mISCU and mISD11 was incubated with 0.1mM ferrous iron sulfate or 1 mM bathophenanthroline disulfonic acid (BP) in the presence of 10mM ascorbic acid (reducing condition) as indicated. Efficient Fe^2+^ chelation by BP was observed as the solution turned red, accounting for the BP-Fe^2+^ complex formation. Purified GST or GST-mFXN was then added to each sample and further incubated to allow GST-mFXN/ISCU/NFS1/ISD11 complex formation. The samples were analysed by native PAGE and coomassie blue staining and by western blot using a frataxin antibody (IB). The ratio between frataxin as a monomer and frataxin in the complex was not modified by iron excess or depletion, indicating that the complex formation is not iron-dependent. (D) Effect of different metals on the complex formation. A bacterial soluble extract containing mNFS1, mISCU and mISD11 was incubated with 0.05mM nickel sulfate, zinc chloride, calcium chloride or magnesium sulfate as indicated. Purified GST or GST-mFXN was then added to each sample and further incubated to allow GST-mFXN/ISCU/NFS1/ISD11 complex formation followed by a GST tag purification. The samples were then analysed by native and denaturing PAGE and coomassie blue staining.
(JPG)

Figure S5 **Characterization of fibroblast cell clones carrying wild type or mutant FXN.** (A) Transient transfection of wild-type FXN or mutants N146A, N146K, W155R and W155A in COS-1 cells. Total extracts were loaded on a SDS-gel and analyzed by Western blot using anti-frataxin and anti-tubulin antibodies. For each construct, signals for precursor, intermediate and mature frataxin are equivalent suggesting that the mutations do not disturb the expression and the maturation process of the protein. NT corresponds to non-transfected COS-1 cells. (B) Genotyping on heterozygous L3/L- cell populations expressing either wild type or mutant (N146A, N146K or mito81–210) hFXN before (L3/L−) and after pEGFP-Cre transfection and clonal sorting. One clone is presented for each construct. (C) Summary of the number of clones obtained for each frataxin mutant and the corresponding cellular and biochemical phenotypes observed. (D) Determination of oxidative stress sensitivity of wild-type FXN and mutants N146A and mito81–210 clones. Cells were incubated with DHR123 and analyzed by FACS. The thin curves represent the autofluorescence of cells without DHR123 treatment. The black curves represent the fluorescence observed with no exogenous stress, and the grey curves represent the fluorescence induced after hydrogen peroxide treatment (20 µM; 30 min). Experiments were done in duplicate on 4 clones of FXN, 3 clones of N146A and 4 clones of mito81–210. I154F mutant clone was used as a positive control. (E) Ultrastructural alterations in the N146K clone observed by electron microscopy analysis. mt, mitochondria; Lp, lipid droplet; mt-Fe, intramitochondrial iron deposits; N, nucleus. (F) Transient transfection of wild-type hFXN or mito81–210 in COS-1 cells. Total extracts (left panel) were loaded on a SDS-gel and analyzed by Western blot using anti-frataxin antibody. NT corresponds to non-transfected COS-1 cells. On the right panel, cellular fractioning of COS-1 cells transfected with mito81–210. Cytosolic and mitochondrial fractions were loaded on gel and analyzed using anti-frataxin antibody by Western blot.
(JPG)

Figure S6 **Purification and characterisation of the FXN/NFS1/ISCU/ISD11 complex.** (A) GST-mFXN was co-expressed with mISCU, HIS-mNFS1 and mISD11 followed by double purification using GSH and nickel columns. The presence of the HIS tag on NFS1 had no effect on complex formation (first and second lanes). The lane on the right shows the complex obtained after co-expression, and purification using the HIS tag of FXN-HIS with mISCU, mNFS1 and mISD11 on a native gel. (B) Gel filtration with the GST-mFXN/ISCU/HIS-NFS1/ISD11 complex. After purification using GSH and Nickel columns, the sample was loaded and separated by gel filtration. Sixty fractions were collected and fractions 2 to 40 were run on SDS- and native-PAGE to determine the protein composition by coomassie blue staining. Fractions 14 to 18 that contained the complex were concentrated for the native mass spectrometry analysis. Note that the signal observed for GST-mFXN after the double purification is due to contamination on the nickel column due to the large excess of GST-mFXN monomer present in the sample before gel filtration. (C) ESI native mass spectrum of the complex with GST-mFXN. The complex was purified as in indicated in (A) and (B) and submitted to native mass spectrometry analysis. The experimental molecular weight for each component obtained in denaturing conditions were 10,722 Da, 14,576 Da, 41,047 Da and 45,553 Da for mISD11, mISCU, GST-mFXN and HIS-mNFS1, respectively. A significant set of peaks (corresponding to the multicharged ions) was detected corresponding to a molecular weight of 237,130 Da. (D) Stoichiometry of the quaternary complex. Comparison of the molecular weight (MW) obtained with different combinations using the observed mass of each component and the observed mass of the complexes. NFS1 was considered as a dimer, by analogy with the bacterial IscS and NifS. The observed mass difference between the GST-mFXN/mISCU/HIS-mNFS1/mISD11 complex and the mFXN-HIS/mISCU/mNFS1/mISD11 complex indicates that there are 2 frataxins per complex. The ISCU∶frataxin ratio is 1 as measured in (F). The residual mass can be attributed to the presence of 3 or 4 ISD11 per complex. (E) Determination of the ISCU∶frataxin ratio within the complex. mISCU-HIS and mFXN-HIS were purified by tag purification and gel filtration (S75). mISCU-HIS and mFXN-HIS fractions were quantified using the theoretical extinction coefficient at 280 nm of 9,970 M^−1^ cm^−1^ and 26,930 M^−1^ cm^−1^, respectively (http://expasy.org/tools/protparam.html). Both fractions were then used to generate standard curves. mFXN-HIS/mISCU/mNFS1/mISD11 complex was purified by coupling affinity tag purification and gel filtration (S200) and loaded on a SDS gel. The quantity of frataxin and mISCU was determined by densitometry after coomassie blue staining, using a Chemi genius^2^ bio imaging system and GeneTools software (Syngene). The result is presented as the average obtained from six independent experiments ± SD. Note that on the rare occasions when frataxin was less expressed, the quantity of frataxin per complex was lower.
(JPG)

Figure S7 **Purification of the mISCU/mNFS1/mISD11 ternary complex.** (A) The mISCU-HIS/mNFS1/mISD11 complex was purified from co-expressing bacteria using HIS-tag purification and gel filtration. Both the eluate (E) after HIS-tag purification (left panel) and the fractions of the gel filtration (right panel) were loaded on SDS-PAGE and analyzed by coomassie blue staining. The ternary complex was isolated in fractions 5 to 7. (B) The mISCUD72A-HIS/mNFS1/mISD11 complex was purified as in (A). The eluate (E) from the HIS-tag purification (left panel) and the fractions of the gel filtration (right) panel were loaded on SDS-PAGE and analyzed by coomassie blue staining. More ternary complex could be obtained compared to wild type mISCU (see (A)) in fractions 5 to 7, indicating that the D72A mutation increases the affinity of mISCU for mNFS1/mISD11.
(JPG)

Materials and Methods S1 (DOC)

Table S1 (XLS)
