# Supplementary figures and images for: Mammalian Frataxin: An Essential Function for Cellular Viability through an Interaction with a Preformed ISCU/NFS1/ISD11 Iron-Sulfur Assembly Complex
Source: PLoS One. 2011 Jan 26;6(1):e16199. doi: 10.1371/journal.pone.0016199 (PMC3027643; doi:10.1371/journal.pone.0016199)

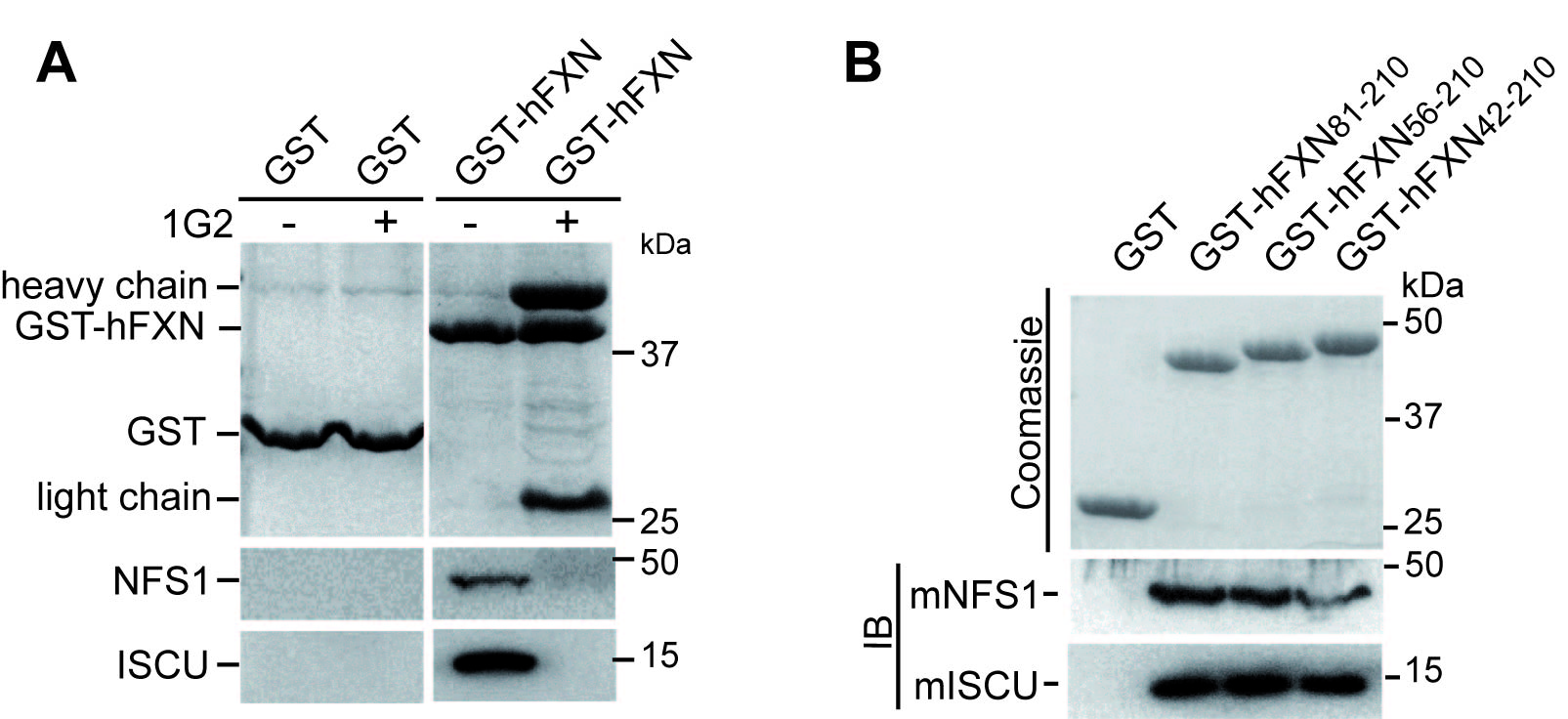

Supplement: Figure S1 — Conserved domain of frataxin is involved in the interaction with ISCU and NFS1. (A) The monoclonal 1G2 antibody directed against exon 3–4 of frataxin prevented the interaction between frataxin and ISCU and NFS1. GST pull-down was performed as described in Fig. 1C with the addition (+) or not (−) of 1G2 antibody. Note that the 1G2 antibody is retained by GST-hFXN (heavy and light chain) and prevents interaction with ISCU and NFS1. (B) GST-hFXN pull-down was performed on mitochondrial Hela extract as in Fig. 1C using different GST-hFXN constructs. As the capacity of GST-hFXN42–210, GST-hFXN56–210 and GST- hFXN81–210 to interact with NFS1 and ISCU were equivalent, we perform all following GST pull-down with the GST-hFXN81–210 expressing construct that corresponds to the size of the endogenous mature human frataxin. (JPG) [file pone.0016199.s001.jpg]

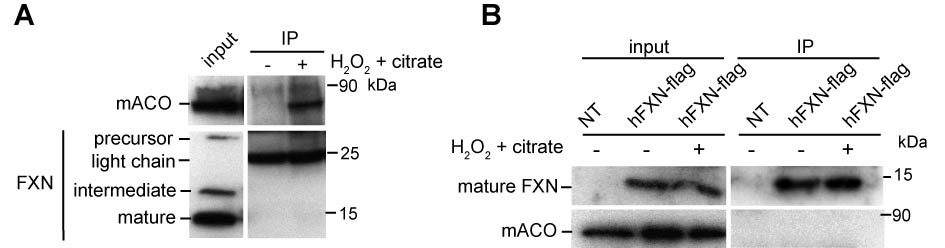

Supplement: Figure S2 — Absence of interaction between frataxin and mitochondrial aconitase. (A) Immunoprecipitation of mitochondrial aconitase from HeLa mitochondrial extracts was performed exactly as described (1), with the same anti-aconitase antibody kindly provided by Anne-Laure Bulteau. Mitochondria were treated with 100 µM hydrogen peroxide and 2 mM of citrate (+) prior to the IP. Samples were loaded on SDS-PAGE and analysed by Western blot using specific antibodies against frataxin and mitochondrial aconitase. (B) Immunoprecipitation of hFXN-FLAG was performed as in Fig. 1A. Mitochondria were treated or not with hydrogen peroxide and citrate as in (A). Samples were analysed by Western blot. (JPG) [file pone.0016199.s002.jpg]

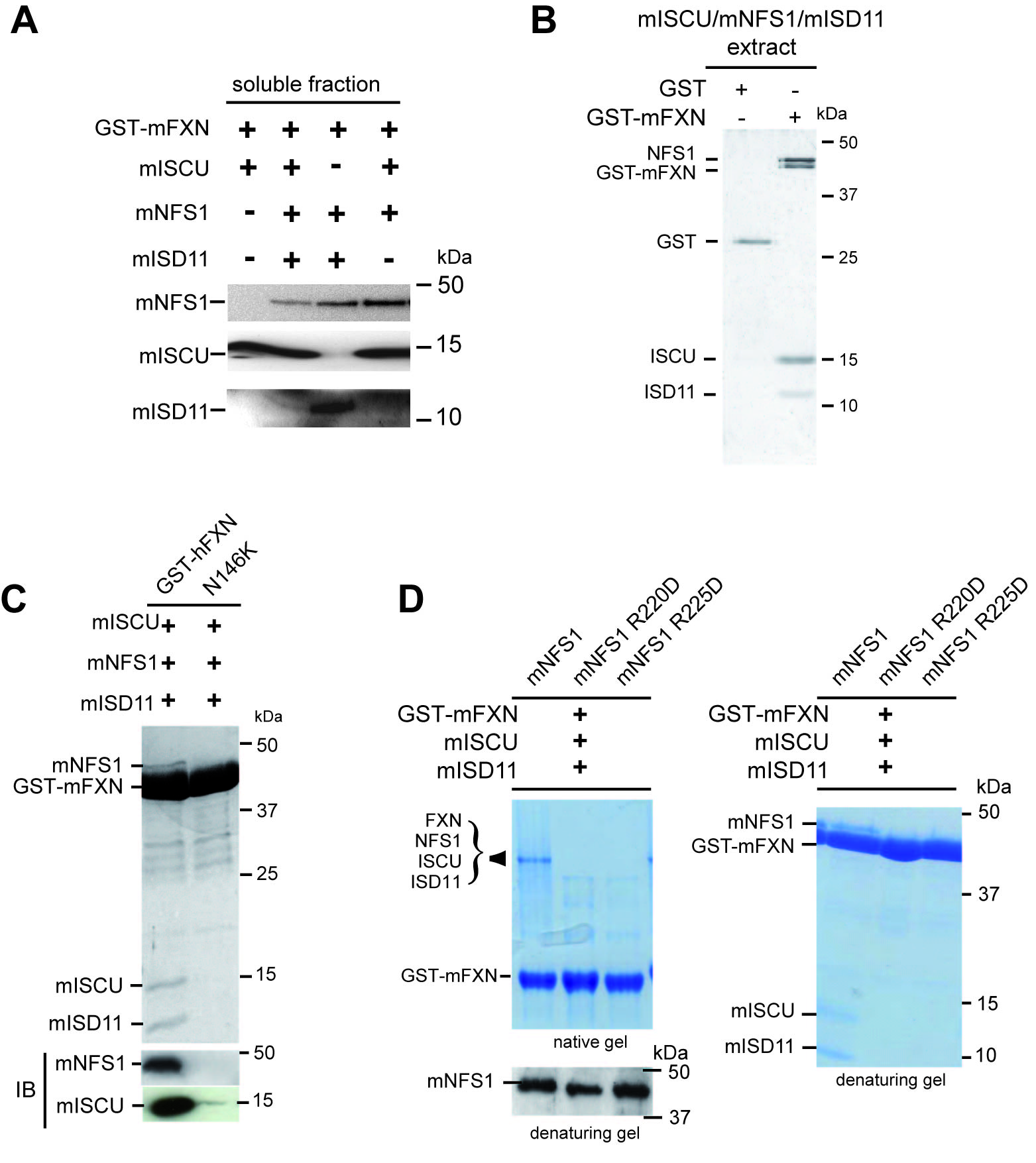

Supplement: Figure S3 — Frataxin interaction with ISCU/NFS1/ISD11 complex and effects of mutations. (A) Expression of mNFS1, mISCU and mISD11 in bacteria co-transformed with different sets of vectors. Soluble fractions of bacteria expressing GST-FXN, mNFS1, mISD11 and/or mISCU were loaded on a SDS-gel and analysed by Western blot using NFS1, ISD11 and ISCU specific antibodies. + and − indicate the presence and the absence of the corresponding vectors, respectively. (B) GST pull-down using a limiting amount of GST-FXN and a bacterial extract expressing mISCU/mNFS1/mISD11. 25mg of GST or GST-FXN were added to the bacterial lysate before purification on glutathione-sepharose beads. The elutions were analyzed on SDS-PAGE by silver staining. (C) Co-purification of ISCU/NFS1/ISD11 with GST-hFXNN146K. GST-hFXN or GST-hFXNN146K (N146K) were co-expressed with mISCU, mNFS1 and mISD11 and purified on glutathione-S-sepharose column as in Fig. 2A. Elutions were analysed by SDS-PAGE and coomassie blue staining (upper panel) or Western blot (IB). (D) Mutations of positively charged residues on NFS1 affect frataxin interaction with the complex. R220D and R225D mutations were introduced by directed mutagenesis on mNFS1 cDNA. Co-purification was carried out as in Fig. 2A and analyzed on native and denaturing gel by coomassie blue staining. Western blot on mNFS1 was performed to verify the correct expression of the two mNFS1 mutants. (JPG) [file pone.0016199.s003.jpg]

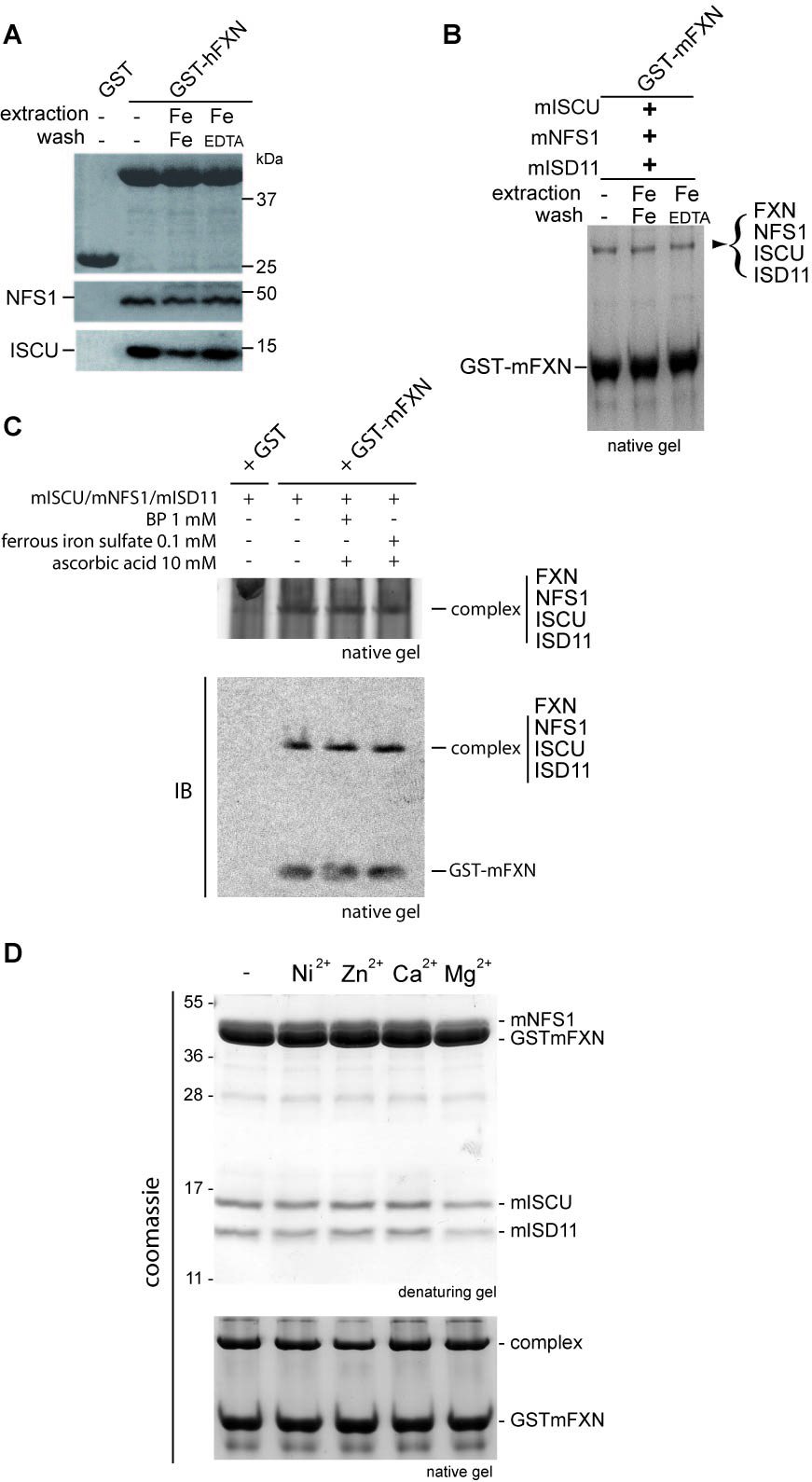

Supplement: Figure S4 — Absence of effect of different metals on complex formation. (A) Effect of iron on the interaction of frataxin with ISCU and NFS1. GST pull-down was carried out as in Fig. 1C with the exception that the extraction, purification and wash steps were carried out in the absence or the presence of 100 mM FeSO4/1 mM ascorbate (Fe2+) and 1 mM EDTA as indicated. (B) Effect of iron on native GST-mFXN/ISCU/NFS1/ISD11 complex. GST-mFXN was co-expressed with mISCU, mNFS1 and mISD11 and purified as in Fig. 2A . Iron or EDTA was added during extraction and wash steps. Samples were loaded on a 7.5% non-denaturing gel and stained with coomassie blue. Western blot analysis and mass spectrometry analysis confirmed that the upper band corresponds to a complex containing mFXN, mISCU, mNFS1 and mISD11. (C) Effect of iron on the complex formation. A bacterial soluble extract containing mNFS1, mISCU and mISD11 was incubated with 0.1mM ferrous iron sulfate or 1 mM bathophenanthroline disulfonic acid (BP) in the presence of 10mM ascorbic acid (reducing condition) as indicated. Efficient Fe2+ chelation by BP was observed as the solution turned red, accounting for the BP-Fe2+ complex formation. Purified GST or GST-mFXN was then added to each sample and further incubated to allow GST-mFXN/ISCU/NFS1/ISD11 complex formation. The samples were analysed by native PAGE and coomassie blue staining and by western blot using a frataxin antibody (IB). The ratio between frataxin as a monomer and frataxin in the complex was not modified by iron excess or depletion, indicating that the complex formation is not iron-dependent. (D) Effect of different metals on the complex formation. A bacterial soluble extract containing mNFS1, mISCU and mISD11 was incubated with 0.05mM nickel sulfate, zinc chloride, calcium chloride or magnesium sulfate as indicated. Purified GST or GST-mFXN was then added to each sample and further incubated to allow GST-mFXN/ISCU/NFS1/ISD11 complex formation followed by a GST tag purifica [file pone.0016199.s004.jpg]

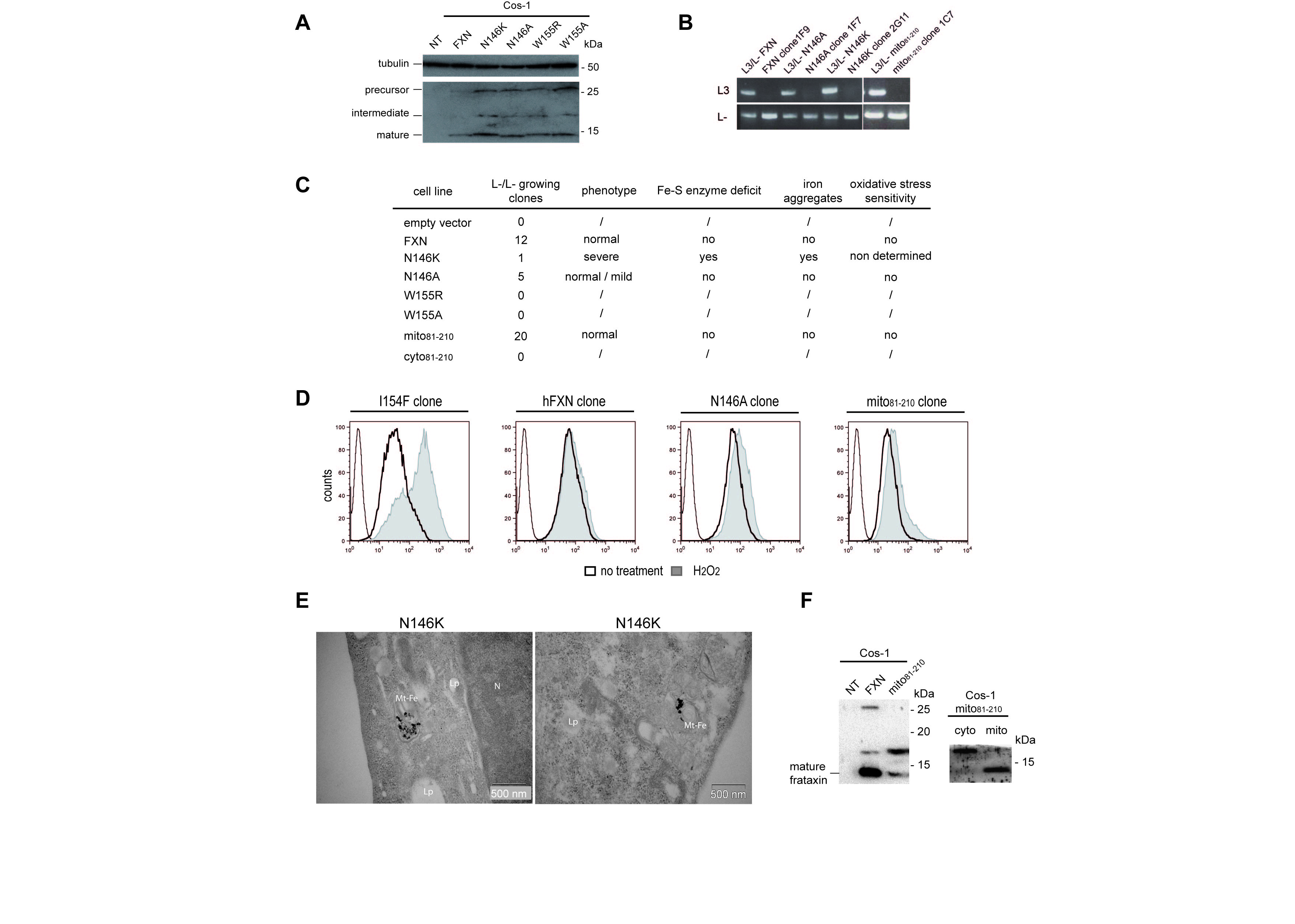

Supplement: Figure S5 — Characterization of fibroblast cell clones carrying wild type or mutant FXN. (A) Transient transfection of wild-type FXN or mutants N146A, N146K, W155R and W155A in COS-1 cells. Total extracts were loaded on a SDS-gel and analyzed by Western blot using anti-frataxin and anti-tubulin antibodies. For each construct, signals for precursor, intermediate and mature frataxin are equivalent suggesting that the mutations do not disturb the expression and the maturation process of the protein. NT corresponds to non-transfected COS-1 cells. (B) Genotyping on heterozygous L3/L- cell populations expressing either wild type or mutant (N146A, N146K or mito81–210) hFXN before (L3/L−) and after pEGFP-Cre transfection and clonal sorting. One clone is presented for each construct. (C) Summary of the number of clones obtained for each frataxin mutant and the corresponding cellular and biochemical phenotypes observed. (D) Determination of oxidative stress sensitivity of wild-type FXN and mutants N146A and mito81–210 clones. Cells were incubated with DHR123 and analyzed by FACS. The thin curves represent the autofluorescence of cells without DHR123 treatment. The black curves represent the fluorescence observed with no exogenous stress, and the grey curves represent the fluorescence induced after hydrogen peroxide treatment (20 µM; 30 min). Experiments were done in duplicate on 4 clones of FXN, 3 clones of N146A and 4 clones of mito81–210. I154F mutant clone was used as a positive control. (E) Ultrastructural alterations in the N146K clone observed by electron microscopy analysis. mt, mitochondria; Lp, lipid droplet; mt-Fe, intramitochondrial iron deposits; N, nucleus. (F) Transient transfection of wild-type hFXN or mito81–210 in COS-1 cells. Total extracts (left panel) were loaded on a SDS-gel and analyzed by Western blot using anti-frataxin antibody. NT corresponds to non-transfected COS-1 cells. On the right panel, cellular fractioning of COS-1 cells transfected with mito81–210. Cyto [file pone.0016199.s005.jpg]

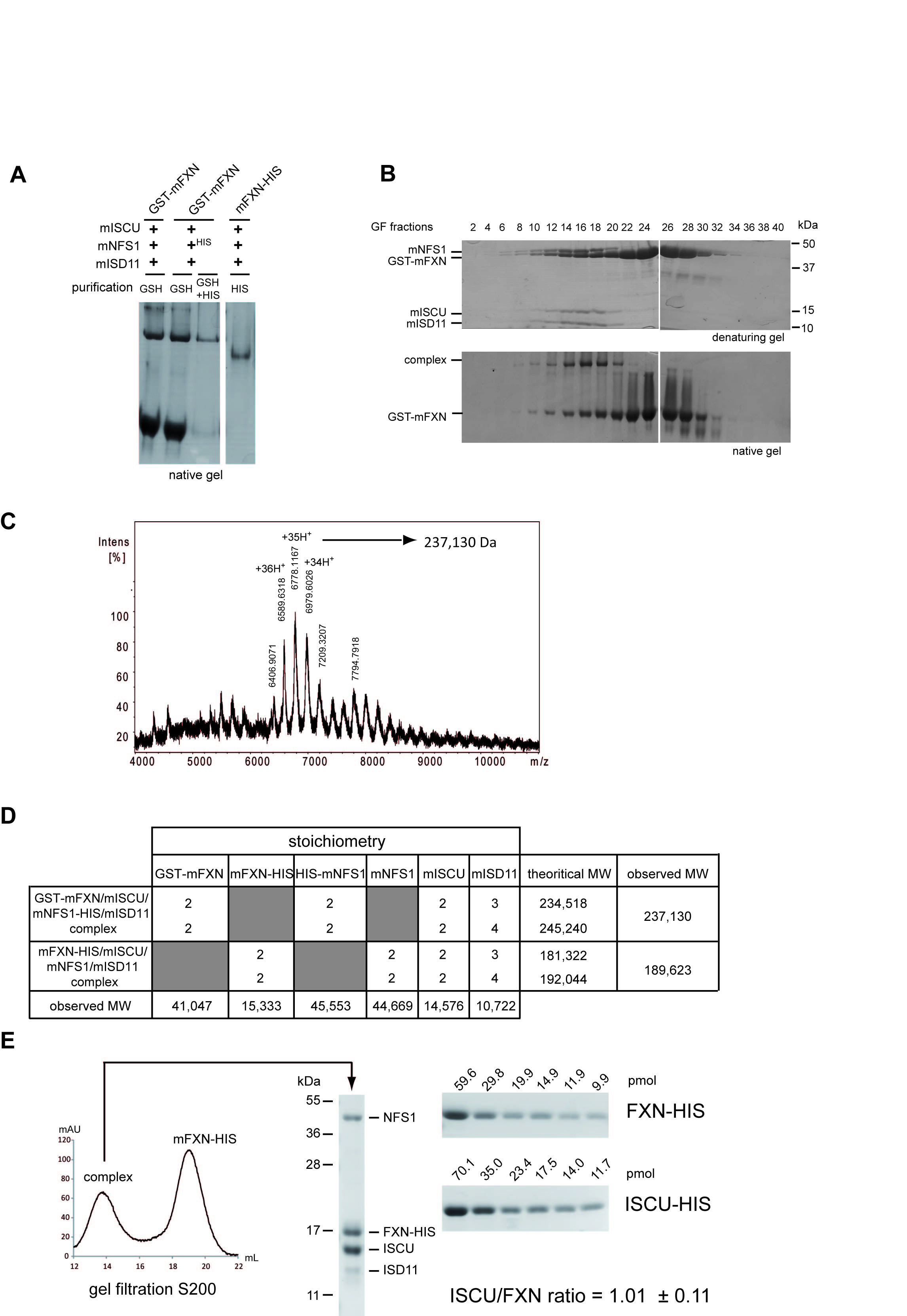

Supplement: Figure S6 — Purification and characterisation of the FXN/NFS1/ISCU/ISD11 complex. (A) GST-mFXN was co-expressed with mISCU, HIS-mNFS1 and mISD11 followed by double purification using GSH and nickel columns. The presence of the HIS tag on NFS1 had no effect on complex formation (first and second lanes). The lane on the right shows the complex obtained after co-expression, and purification using the HIS tag of FXN-HIS with mISCU, mNFS1 and mISD11 on a native gel. (B) Gel filtration with the GST-mFXN/ISCU/HIS-NFS1/ISD11 complex. After purification using GSH and Nickel columns, the sample was loaded and separated by gel filtration. Sixty fractions were collected and fractions 2 to 40 were run on SDS- and native-PAGE to determine the protein composition by coomassie blue staining. Fractions 14 to 18 that contained the complex were concentrated for the native mass spectrometry analysis. Note that the signal observed for GST-mFXN after the double purification is due to contamination on the nickel column due to the large excess of GST-mFXN monomer present in the sample before gel filtration. (C) ESI native mass spectrum of the complex with GST-mFXN. The complex was purified as in indicated in (A) and (B) and submitted to native mass spectrometry analysis. The experimental molecular weight for each component obtained in denaturing conditions were 10,722 Da, 14,576 Da, 41,047 Da and 45,553 Da for mISD11, mISCU, GST-mFXN and HIS-mNFS1, respectively. A significant set of peaks (corresponding to the multicharged ions) was detected corresponding to a molecular weight of 237,130 Da. (D) Stoichiometry of the quaternary complex. Comparison of the molecular weight (MW) obtained with different combinations using the observed mass of each component and the observed mass of the complexes. NFS1 was considered as a dimer, by analogy with the bacterial IscS and NifS. The observed mass difference between the GST-mFXN/mISCU/HIS-mNFS1/mISD11 complex and the mFXN-HIS/mISCU/mNFS1/mISD11 complex indicates t [file pone.0016199.s006.jpg]

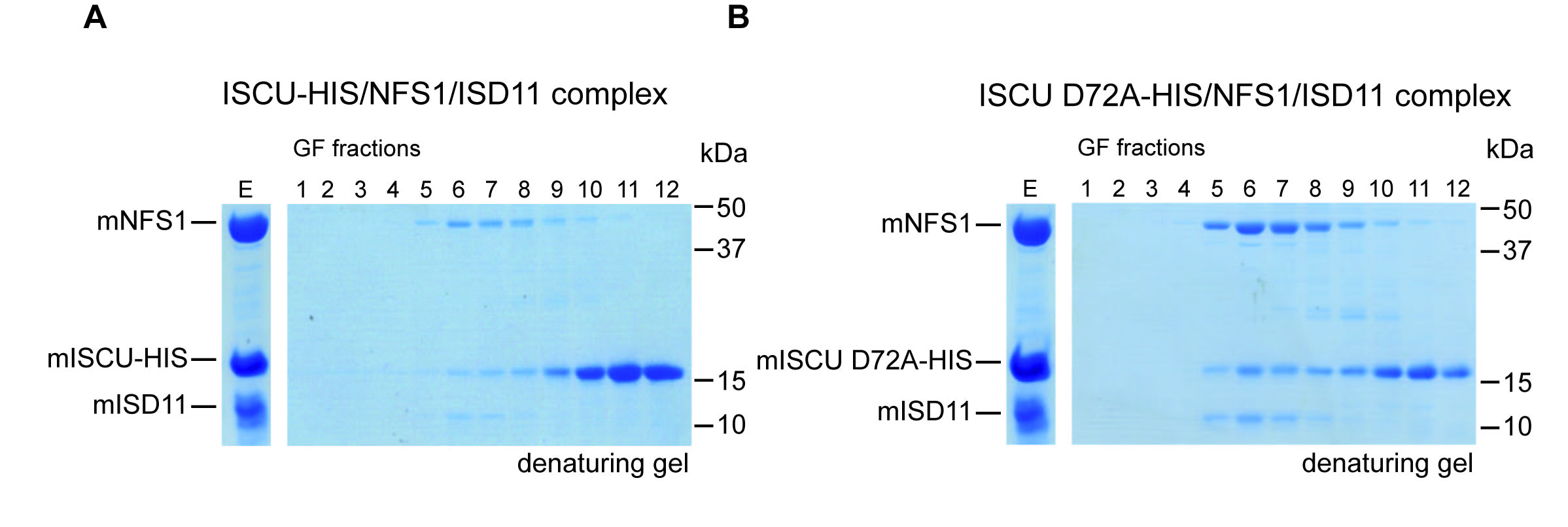

Supplement: Figure S7 — Purification of the mISCU/mNFS1/mISD11 ternary complex. (A) The mISCU-HIS/mNFS1/mISD11 complex was purified from co-expressing bacteria using HIS-tag purification and gel filtration. Both the eluate (E) after HIS-tag purification (left panel) and the fractions of the gel filtration (right panel) were loaded on SDS-PAGE and analyzed by coomassie blue staining. The ternary complex was isolated in fractions 5 to 7. (B) The mISCUD72A-HIS/mNFS1/mISD11 complex was purified as in (A). The eluate (E) from the HIS-tag purification (left panel) and the fractions of the gel filtration (right) panel were loaded on SDS-PAGE and analyzed by coomassie blue staining. More ternary complex could be obtained compared to wild type mISCU (see (A)) in fractions 5 to 7, indicating that the D72A mutation increases the affinity of mISCU for mNFS1/mISD11. (JPG) [file pone.0016199.s007.jpg]
