# Supplementary material for: Mammalian Frataxin: An Essential Function for Cellular Viability through an Interaction with a Preformed ISCU/NFS1/ISD11 Iron-Sulfur Assembly Complex
Source: PLoS One. 2011 Jan 26;6(1):e16199. doi: 10.1371/journal.pone.0016199 (PMC3027643; doi:10.1371/journal.pone.0016199)
Supplement: Materials and Methods S1 — (DOC) [file pone.0016199.s008.doc]

**SUPPLEMENTARY MATERIAL AND METHODS S1**

**Expression plasmids and mutagenesis.** For mammalian expression, hFXN or hFXN-FLAG were cloned between EcoRI and XhoI sites of pcDNA3.1(+) vector. Cloning of mito81-210 into pcDNA3.1(+) vector was performed by PCR amplifying fragment 1-41 and 81-210 of hFXN separately and by introducing a XbaI restriction site for ligation. hFXN (aa 81-210) and mFXN (aa 76-207) were cloned into pGEX4T1 vector using EcoRI and XhoI restriction sites. For bacterial co-expression, mISCU and mFXN were cloned into pACYCDuet-1 or pETDuet-1 vectors using NcoI and XhoI restriction sites. mNFS1 cDNA, without the predicted mitochondrial targeting sequence, was cloned into pENTR-1 vector using EcoRI and XhoI restriction sites and was then subcloned, using Gateway® technology, into pCo0GWS, containing the mISD11 cDNA between the NdeI and BamHI restriction sites. Mutagenesis was performed by PCR directed mutagenesis as previously described [1]. All constructs were verified by sequencing.

**Recombinant protein expression and purification*.*** Transformed BL21 bacteria were cultured in 2xYT in the presence of the relevant antibiotic to reach 0.5 O.D._600_. IPTG 0.1-0.5mM was added to induce recombinant protein production for 3-8 hours. The bacterial pellet was resuspended in cold PBS-TND (PBS, 1% Triton X-100, 1% IGEPAL, DTT 10mM and Complete protease inhibitor cocktail (Roche)), sonicated 5 min at 4°C (amplitude 30%), and incubated 15 min on ice.

**Antibody dilutions.** anti-frataxin 1/1,000 (R1250), anti-β-tubulin 1/10,000 (IGBMC), anti-aconitase 1/10,000 (R2377), anti-ferrochelatase 1/1,000 (R2381), anti-MnSOD 1/5,000 (SOD-110, Stressgen), anti-NFS1 1/2,000 (kindly provided by Cécile Bouton), anti-ISCU 1/2,000 (R2385, against peptide CKLQIQVDEKGKIVDARFK), anti-ISD11 1/5,000 (kindly provided by Cécile Bouton). HRP-coupled secondary antibodies were diluted at 1/5,000.

**Mass spectrometry analysis.** NanoLC-nanoESI/MS^2^ was performed on a linear ion trap LTQ XL (Thermo FisherScientific) with nano-ESI source interfaced to an Ultimate 3000 nanoLC system (Dionex). Samples were first desalted and concentrated on a reverse phase precolumn 300 μm i.d x 5 mm for 3 min at 20 μL/min. Protein digests were then separated on a Pepmap C18, 15 cm x 75 μm i.d (Dionex). The HPLC gradient was 5-60% solvent B (A = 5% acetonitrile, 0.1% formic acid; B = 80% acetonitrile, 0.08% formic acid) in 40 min at a flow rate of 200 nL/min. MS/MS spectra were recorded in the data dependent mode on the five most intense ions observed in MS scan recorded in the mass range m/z 400-1600, the enhanced scan rate was used for the full MS spectrum. A micro zoom scan was acquired before MS/MS acquisition scans to determine the peptide charge. Parameters for acquiring CID MS/MS spectra were as follows; activation time = 30 ms, activation Q = 0.25, relative collision energy = 35% and an isolation width of 3 m/z. Peptides selected for MS/MS acquisition were then placed on an exclusion list for 30 s to limit duplicate spectra. Proteome Discoverer 1.1 (Thermo Fisher Scientific) with Sequest® search engine was used for spectra preprocessing protein ID. The searches were conducted against the human Swissprot database (version 57.9, 512,205 entries). Database searches were performed with the following fixed parameters; precursor mass tolerance of +/- 0.5 Da, product ion mass tolerance +/- 0.8 Da, and 2missed cleavages, carbamidomethylation of cysteine as fixed modification and methionine oxidation as variable modification. Sequest® results were filtered with Xcorr versus charge state 0.7-1, 2.2-2, 2.5-3, 2.75-4.

ESI-MS (electrospray ionisation mass spectrometry) mass spectra were acquired on a time-of-flight mass spectrometer (MicrOTOF II, Bruker Daltonic) equipped with an electrospray source. Calibration of the instrument from m/z 4000-15000 was performed using the cesium iodide (CsI) clusters generated by spraying a solution of CsI in water/2-propanol 50/50 v/v at a concentration of 1 mg/mL. The sample was infused in the mass spectrometer via a syringe pump (kdScientific) at a flow rate of 3 µL/min. The mass spectrometer was controlled via the MicrOTOF control software (version 2.3). The voltage applied was 4.5 kV. The nebulizer gas pressure was 8.7 psi. The source temperature was kept at 160°C. All measurements were performed in the positive ion mode. The optimal instrumental conditions to observe protein complexes were capillary exit voltage 200 V, skimmer1 35 V, hexapole1 21 V, hexapole RF 800 Vpp. Data were acquired for 1 minute. Before data processing, each spectrum was smoothed (Gauss smooth, 0.3 Da) with the DataAnalysis software (version 4.0).

1. Schmucker, S., et al. (2008) The in vivo mitochondrial two-step maturation of human frataxin *Hum Mol Genet* 17, 3521-31.
